# Supplementary material for: Exhaustive identification of genome-wide binding events of transcriptional regulators
Source: Nucleic Acids Res. 2024 Mar 18;52(7):e40. doi: 10.1093/nar/gkae180 (PMC11040144; doi:10.1093/nar/gkae180)
Supplement: gkae180_Supplemental_Files [file gkae180_supplemental_files.zip › Supplementary_Figures_Thirdrevision.pdf]

Figure S1

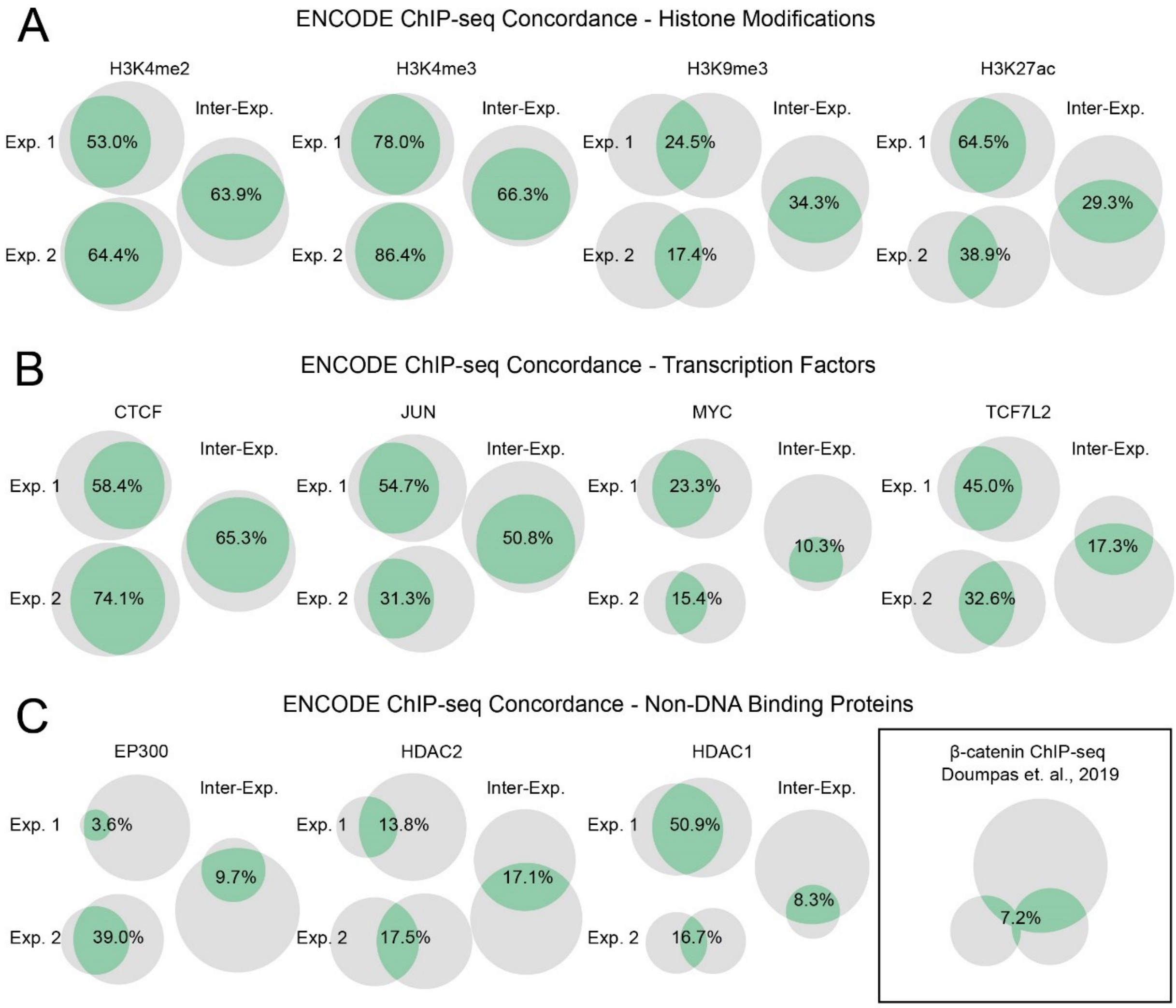

Figure S1. ChIP-seq replicates display low concordance. Proportional Venn diagrams showing peak concordance (green sections) across published ChIP-seq datasets for histone modifications (**A**), transcription factors (**B**) and non-DNA-binding transcriptional regulators and associated proteins (**C**). Concordant peaks are shown as the percentage of total peaks called. For each protein target, we selected datasets that comprise at least two ChIP-seq experiments, each performed at least in technical duplicate. For each target, the Venn diagrams on the left show the concordance between technical duplicates (Exp.1: replicate 1 vs replicate 2; Exp. 2: replicate 1 vs replicate 2). These concordant peaks were then selected and compared cross-experiment and represented in the Venn diagram on the right (Exp 1 vs Exp 2). Notice that β-catenin ChIP-seq (Doumpas et al. 2019) was performed in triplicate with three different anti-β-catenin antibodies.

Figure S2

β-catenin CUT&RUN-LoV-U, 25 replicates

Sequencing depth - peaks - FrIP score

Sample ID	Sequencing Depth (reads)	Peaks (MACS q05)	FrIP Score
19	4475982	561	1.6998
4	4652756	2481	2.7640
6	5104693	882	1.7918
1	5147800	155	1.0902
10	5179425	210	1.6682
20	5209651	188	1.1861
13	5269789	2403	2.2789
17	5497036	424	1.4297
12	5717921	1002	1.7294
15	5820536	889	1.6314
25	5831299	52	0.9766
2	5945031	653	1.5428
18	6355111	253	1.2025
9	6360157	451	1.3466
22	6478777	95	1.0362
11	6836494	1580	1.6682
8	6922514	521	1.3240
24	6943774	124	1.0273
23	7101518	531	1.3124
3	7342648	1048	1.6169
21	7362426	188	1.1307
16	7513717	1149	1.5751
7	7527757	142	1.0848
14	7563362	5762	2.6110
5	8215173	391	1.2557

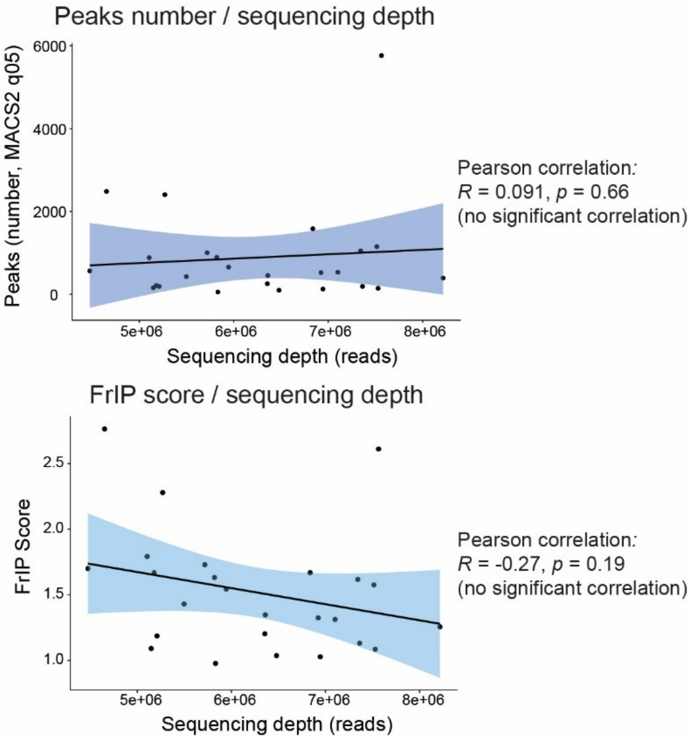

Figure S2. Peaks number or FrIP score do not correlate with sequencing depth among the 25 β-catenin CUT&RUN replicates. Left: table showing sequencing depth (reads), number of peaks called (MACS2 q05) and FrIP score for each of the 25 β-catenin CUT&RUN replicates. Right: plots reporting peak numbers (upper plot) or FrIP score (lower plot) in correlation with sequencing depth. No significant correlation (Pearson correlation) was found between the variables considered.

Figure S3

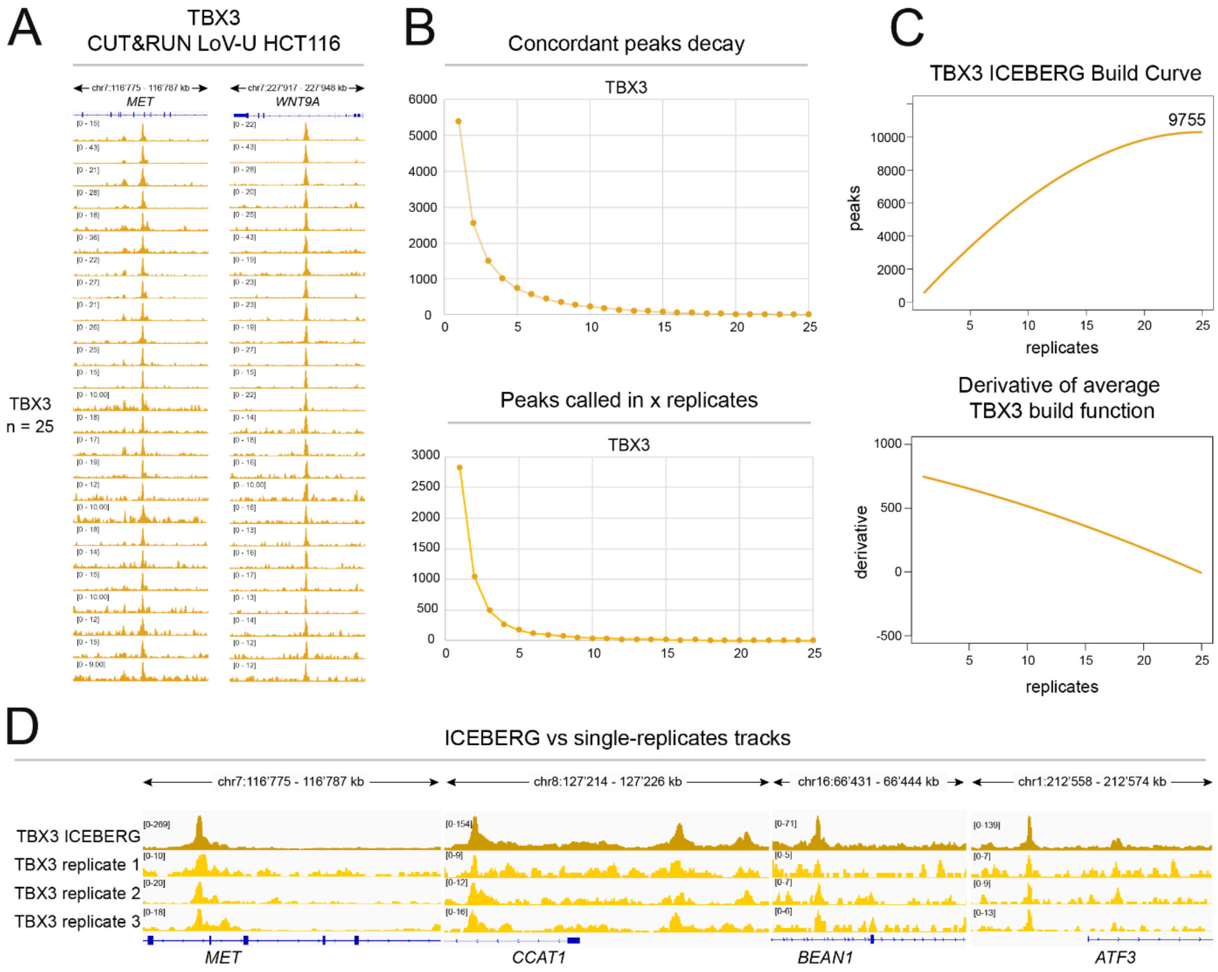

Figure S3. A. Tracks of all performed TBX3 replicates at the positive control *MET* and *WNT9A* loci. All replicates show enrichment and high signal to noise ratio. **B.** Top: decay curve based on the number of peaks called in at least *x* replicates. The curve of TBX3 is similar to β-catenin (Figure 2D). Bottom: decay curve based on the number of peaks called in exactly *x* datasets. **C.** Top: Regression curve describing the number of called peaks per each added replicate during the ICEBERG aggregate generation. Bottom: Plot of the first derivates of the ICEBERG regression curve. **D.** Example comparisons of the ICEBERG dataset versus individual replicates on the loci *MET, CCAT1, BEAN1* and *ATF3.*

Figure S4

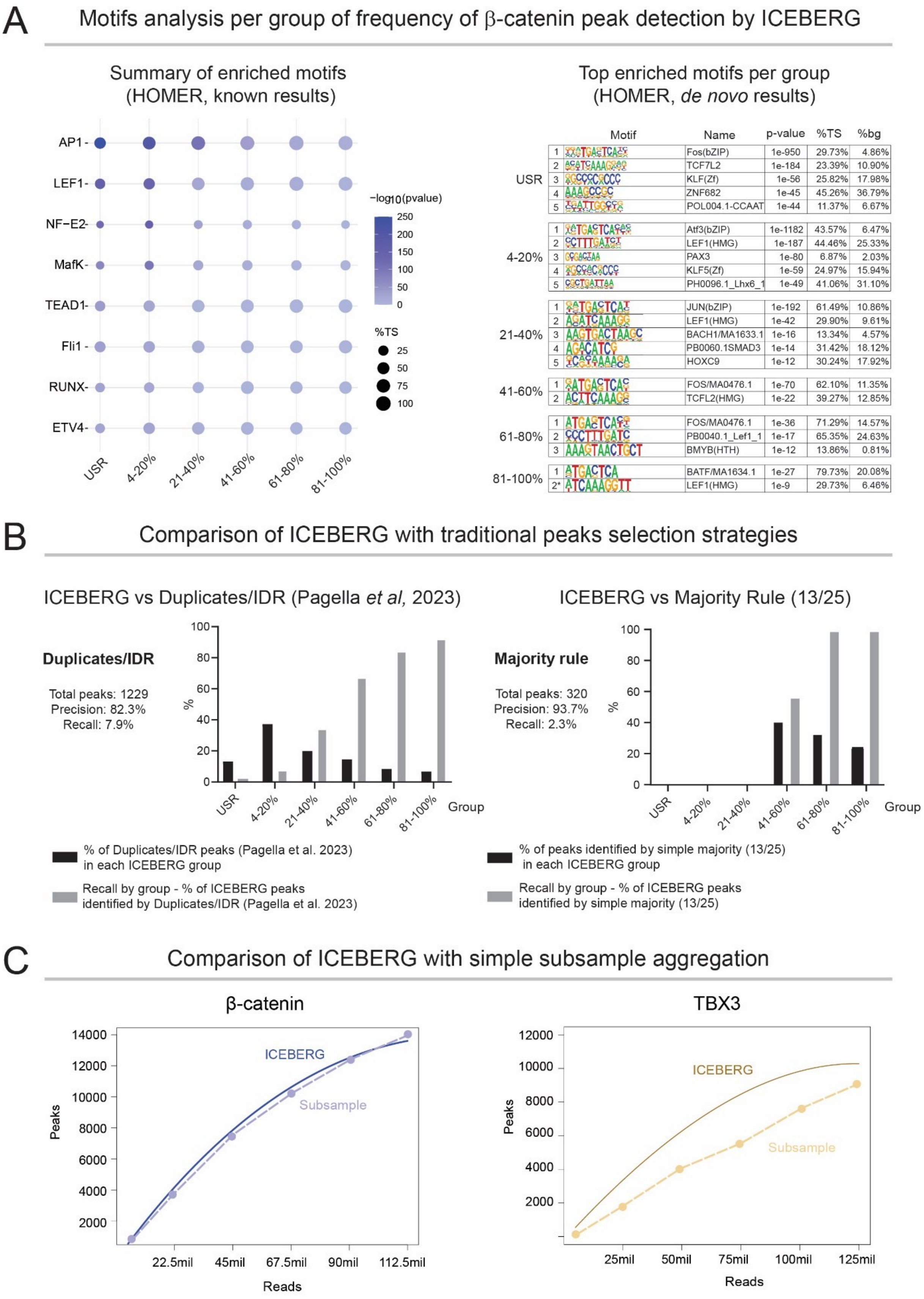
A
Motifs analysis per group of frequency of β-catenin peak detection by ICEBERG
Summary of enriched motifs (HOMER, known results)
AP1
LEF1
NF−E2
MafK
TEAD1
Fli1
RUNX
ETV4
USR
4-20%
21-40%
41-60%
61-80%
81-100%
−log10(pvalue)
250
200
150
100
50
0
%TS
25
50
75
100
Top enriched motifs per group (HOMER, de novo results)
Motif
Name
p-value
%TS
%bg
USR
1 Fos(bZIP) 1e-950 29.73% 4.86%
2 TCF7L2 1e-184 23.39% 10.90%
3 KLF(Zf) 1e-56 25.82% 17.98%
4 ZNF682 1e-45 45.26% 36.79%
5 POL004.1-CCAAT 1e-44 11.37% 6.67%
4-20%
1 Atf3(bZIP) 1e-1182 43.57% 6.47%
2 LEF1(HMG) 1e-187 44.46% 25.33%
3 PAX3 1e-80 6.87% 2.03%
4 KLF5(Zf) 1e-59 24.97% 15.94%
5 PH0096.1_Lhx6_1 1e-49 41.06% 31.10%
21-40%
1 JUN(bZIP) 1e-192 61.49% 10.86%
2 LEF1(HMG) 1e-42 29.90% 9.61%
3 BACH1/MA1633.1 1e-16 13.34% 4.57%
4 PB0060.1SMAD3 1e-14 31.42% 18.12%
5 HOXC9 1e-12 30.24% 17.92%
41-60%
1 FOS/MA0476.1 1e-70 62.10% 11.35%
2 TCFL2(HMG) 1e-22 39.27% 12.85%
61-80%
1 FOS/MA0476.1 1e-36 71.29% 14.57%
2 PB0040.1_Lef1_1 1e-17 65.35% 24.63%
3 BMYB(HTH) 1e-12 13.86% 0.81%
81-100%
1 BATF/MA1634.1 1e-27 79.73% 20.08%
2* LEF1(HMG) 1e-9 29.73% 6.46%
B
Comparison of ICEBERG with traditional peaks selection strategies
ICEBERG vs Duplicates/IDR (Pagella et al, 2023)
Duplicates/IDR
Total peaks: 1229
Precision: 82.3%
Recall: 7.9%
%
100
80
60
40
20
0
USR
4-20%
21-40%
41-60%
61-80%
81-100%
Group
% of Duplicates/IDR peaks (Pagella et al. 2023) in each ICEBERG group
Recall by group - % of ICEBERG peaks identified by Duplicates/IDR (Pagella et al. 2023)
ICEBERG vs Majority Rule (13/25)
Majority rule
Total peaks: 320
Precision: 93.7%
Recall: 2.3%
%
100
80
60
40
20
0
USR
4-20%
21-40%
41-60%
61-80%
81-100%
Group
% of peaks identified by simple majority (13/25) in each ICEBERG group
Recall by group - % of ICEBERG peaks identified by simple majority (13/25)
C
Comparison of ICEBERG with simple subsample aggregation
β-catenin
Peaks
14000
12000
10000
8000
6000
4000
2000
ICEBERG
Subsample
22.5mil
45mil
67.5mil
90mil
112.5mil
Reads
TBX3
Peaks
12000
10000
8000
6000
4000
2000
ICEBERG
Subsample
25mil
50mil
75mil
100mil
125mil
Reads

Figure S4. Further characterization of β-catenin ICEBERG peaks identified with different frequencies in individual replicates. **A.** Motifs analysis (HOMER) performed on peaks belonging to the different detection frequency groups. Left: summary of HOMER known results. Right: HOMER *de novo* results, divided per frequency group. Five top-ranked, significant motifs are shown. Notice that LEF1(HMG) within group 81-100% is potentially a false positive (marked with *). **B.** Comparison of β-catenin ICEBERG with traditional peak selection strategies. Left: comparison of β-catenin peaks discovery in HCT116 cells with ICEBERG (this work) and with a duplicates/IDR approach, as described in Pagella et al., 2023, Cell Systems. Most peaks identified by duplicates/IDR are ICEBERG peaks (precision: 82.3%); however, duplicates/IDR detected less than one-tenth of ICEBERG peaks (recall: 7.9%). Duplicates/IDR identified nearly all high-frequency ICEBERG peaks, while it missed most of low-frequency ICEBERG peaks (grey bars in bar-plot). Notably, despite the low recall, most duplicates/IDR peaks belong to the 4-20% frequency group of ICEBERG peaks. Right: comparison of β-catenin peaks discovery in HCT116 cells with ICEBERG (this work) and with a simple majority rule approach (peaks identified in 13 out of 25 replicates, from this work). The majority approach allowed identification of only 320 peaks, with a precision of 93% (93% of majority-approach-peaks are ICEBERG peaks) and recall of only 2.3% (All majority-approach-peaks represent only 2.3% of all ICEBERG peaks). The majority approach identifies all ICEBERG peaks in high-probability groups (61-80% and 81-100%), but none of the low-probability ICEBERG peaks (< 40%). **C.** Graphs comparing the performance of the ICEBERG pipeline (build curves from Figure 3C and S3C) at increasing sequencing depths in comparison to random subsampling from a pool of all sequenced reads from all replicates. For β-catenin (left) where the replicates were relatively homogenous in sequencing depth, ICEBERG performed similarly to random subsampling. For TBX3 (right), which had higher variation in sequencing depths and greater inter-sample variation, ICEBERG recovered more peaks over all tested depths. Abbreviations - %TS: % of target sequences; %bg: % of background sequences; USR: Undetectable in Single Replicates (see Figure 5).
